# Aortoduodenal fistula successfully treated with endovascular repair

**DOI:** 10.1002/ccr3.3641

**Published:** 2020-12-25

**Authors:** Junya Fuchigami, Kyohei Miyamoto, Naoaki Shibata, Takafumi Yonemitsu, Kentaro Ueda, Seiya Kato

**Affiliations:** ^1^ Department of Emergency and Critical Care Medicine Wakayama Medical University Wakayama Japan

**Keywords:** endovascular aortic repair (EVAR), gastrointestinal endoscopy, primary aortoduodenal fistula, upper gastrointestinal bleeding

## Abstract

Aortoduodenal fistula is a rare cause of life‐threatening upper gastrointestinal bleeding. Accurate diagnosis is essential to initiate definitive treatment because endoscopic hemostasis, which is the usual initial intervention for upper gastrointestinal bleeding, may be ineffective. This case underscores timely intervention using endovascular treatment for achieving hemostasis in aortoduodenal fistula.

## Graphical abstract

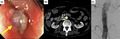

Question: What is the cause of life‐threatening upper gastrointestinal bleeding that cannot be treated with endoscopy?

Answer: Aortoduodenal fistula is an important cause of upper gastrointestinal bleeding that requires timely endovascular treatment, and not endoscopy.

A previously healthy 76‐year‐old man visited the emergency department with massive hematemesis and melena. His blood pressure and heart rate were 152/76 mm Hg and 94 beats per minute, respectively. Laboratory testing revealed hemoglobin level of 7.0 g/dL, international normalized ratio of prothrombin time of 1.33, and activated partial thromboplastin time of 40.4 seconds. Hematemesis persisted, necessitating transfusion of 8 units of red blood cells and 8 units of fresh frozen plasma. Gastrointestinal endoscopy revealed a duodenal ulcer with a pulsating vasculature (Figure 1A). Endoscopic hemostasis was attempted using hemoclips, but proved unsuccessful. Contrast‐enhanced computed tomography revealed an aortoduodenal fistula (Figure 1B). Emergent endovascular aortic repair (EVAR) was performed to resolve hematemesis (Figure 1C). The patient was discharged 2 weeks after EVAR and was found to be healthy during the follow‐up 11 months after discharge.

**FIGURE 1:**
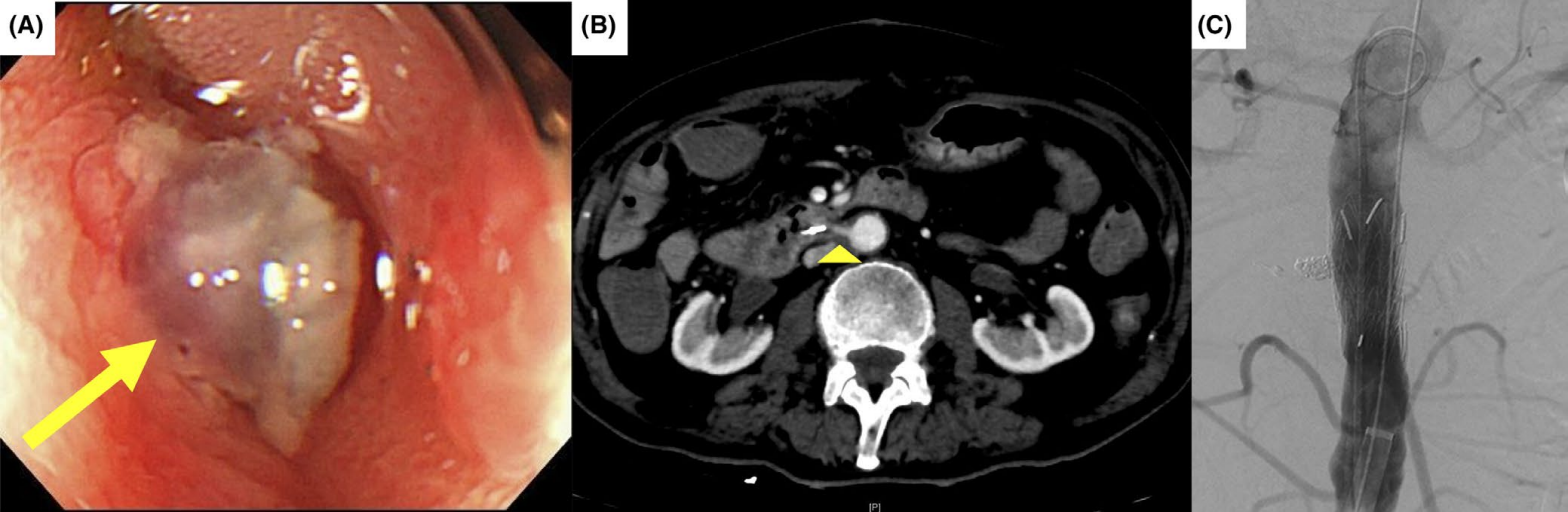
A, Gastrointestinal endoscopy of the duodenum. The endoscopy revealed a duodenal ulcer with a pulsating vasculature (arrow). B, Contrast‐enhanced computed tomography arterial phase. The arterial phase showed the aorta directly communicating with the duodenum (arrowhead). C, Aortography after endovascular aortic repair. Excluder aortic cuff (2.3‐3.3 cm) was used as endovascular aortic graft.

Aortoduodenal fistula is an unusual cause of upper gastrointestinal bleeding.^1^ Despite its rarity, accurate diagnosis is essential to initiate specific and lifesaving treatment. Our case highlights the ineffectiveness of endoscopic treatment and the importance of timely intervention using endovascular treatment for achieving hemostasis in aortoduodenal fistula.^2^

## CONFLICT OF INTEREST

There are no conflicts of interest in all authors about this manuscript.

## AUTHOR CONTRIBUTIONS

JF: wrote the first draft and contributed to the management of the patient and approved the final manuscript. KM, NS, TY, KU, and SK: contributed to the management of the patient, revised the manuscript, and approved the final manuscript.

## ETHICAL APPROVAL

Written informed consent for publication was obtained from the patient.

## INFORMED CONSENT

Consent for publication was obtained from the patient.

## Data Availability

The datasets used and/or analyzed during the current study are available from the corresponding author on reasonable request.
